# A High Percentage of NSCLC With Germline *CHEK2* Mutation Harbors Actionable Driver Alterations: Survey of a Cancer Genomic Database and Review of Literature

**DOI:** 10.1016/j.jtocrr.2022.100387

**Published:** 2022-08-06

**Authors:** Shannon S. Zhang, Jessica K. Lee, Hanna Tukachinsky, Alexa B. Schrock, Misako Nagasaka, Sai-Hong Ignatius Ou

**Affiliations:** a Department of Medicine, University of California Irvine School of Medicine, Orange, California; b Foundation Medicine Inc., Cambridge, Massachusetts; c Chao Family Comprehensive Cancer Center, Orange, California

**Keywords:** Germline *CHEK2* mutation, Actionable driver mutation, NSCLC, EGFR, KRAS, Tumor genotyping, Plasma genotyping

## Abstract

**Introduction:**

Germline *CHEK2* mutations are rare and have not been associated with increased risk of NSCLC.

**Methods:**

We identified two sequential primary NSCLCs harboring distinct actionable driver alterations (EGFR E746 _S752 delinsV and *CD74-ROS1*) in a patient with NSCLC with a novel germline *CHEK2* mutation S5fs∗54 (c.14_20delCGGATGT). We queried a genomic database of NSCLC samples profiled by plasma next-generation sequencing (Foundation Medicine Inc.) and performed a literature search of germline *CHEK2* mutations in NSCLC.

**Results:**

Of 6101 patients with unique NSCLC profiled by plasma next-generation sequencing, 53 cases (0.87%) of germline *CHEK2* mutation were identified (male-to-female ratio, 49%:51%; median age = 75 y). The median allele frequency of *CHEK2* was 49% (interquartile range: 49%–51%). Ten unique *CHEK2* germline mutations were identified. Literature review identified 15 additional cases of germline *CHEK2* mutations in NSCLC. Overall, a total of 70 *CHEK2* germline mutations (21 unique *CHEK2* alterations) were identified. Among these 70 *CHEK2* germline mutations, 54.3% were amino acid substitutions (point mutation), 40.0% were frameshift mutations, and 5.7% were splice site mutations. Of these 70 total cases assessed, 29 (41.4%) potentially actionable driver alterations were identified with *KRAS* G12C mutation (27.6%) being the most common and *KRAS* G12A/C/D/R/S/V mutations together constituting 51.7% of these driver mutations.

**Conclusions:**

Germline *CHEK2* mutations are rare in NSCLC. A large proportion of these cases harbor actionable driver alterations. The relationship between germline *CHEK2* mutations and actionable driver alterations in NSCLC may be worth further investigation.

## Introduction

The *CHEK2* gene encodes checkpoint kinase CHK2, activated mainly in response to DNA double-stranded breaks. There are several germline hotspot mutations (IVS2+1G>A; 1100delC; I157T) in *CHEK2* that are associated with hereditary breast and prostate cancers.^1^ Nevertheless, no germline *CHEK2* (*gCHEK2*) mutations (*gCHEK2m*) have been associated with hereditary lung cancer and there is very limited literature on *gCHEK2m* and NSCLC. During routine clinical care, we identified a patient with NSCLC with a rare *gCHEK2m* who developed two sequential primary lung cancer with different actionable driver alterations, which prompted us to investigate the relationship between *gCHEK2m* and actionable driver alterations in NSCLC.

## Materials and Methods

We queried the Foundation Medicine genomic database of NSCLC plasma samples that were profiled by next-generation sequencing (NGS) to assess frequency of *CHEK2* mutations and any associated actionable driver alterations. *CHEK2* germline status was determined using a previously described research algorithm.^2^ We further performed a PubMed search for *gCHEK2m* in NSCLC.

## Results

**Figure 1:**
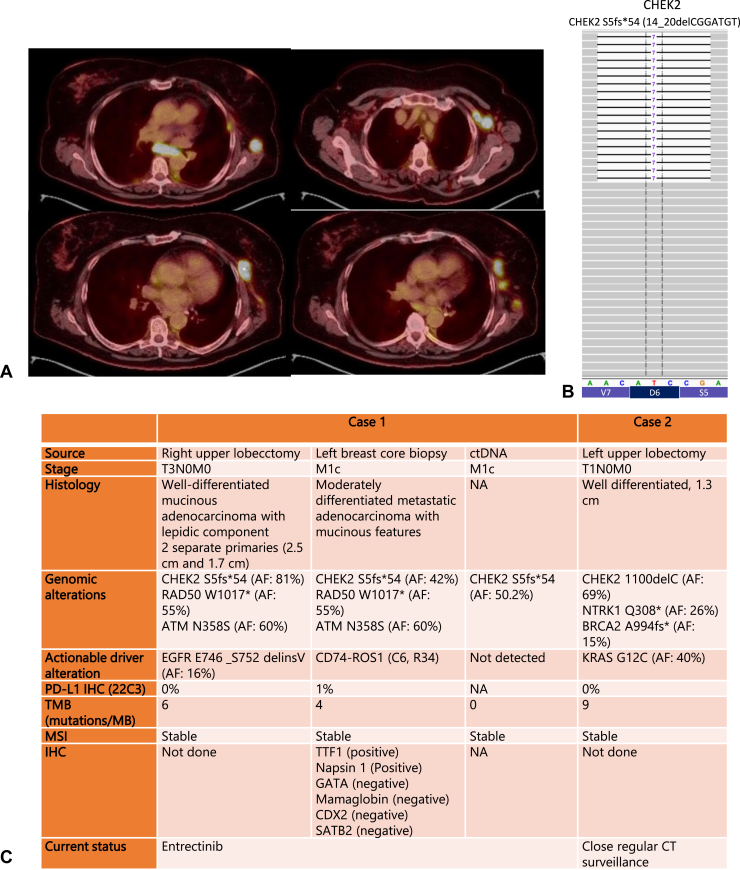
(*A*) PET scan revealing involvement of the *CD74-ROS1 NSCLC*. Left upper scan revealing involvement of the left breast and mediastinal lymphadenopathy. Right upper scan revealing involvement in the left retropectoral region. Lower level scans revealing involvement of the left axillary lymph nodes at multiple levels. (*B*) Integrated genome viewer of the *CHEK2* mutation S5fs∗54 (c.14_20delCGGATGT) of our patient case #1 with an allele frequency of 50.2% in ctDNA. (*C*) Clinical, genomic, and immunohistochemical characteristics of patient cases #1 and #2. #, number; AF, allele frequency; CT, computed tomography; ctDNA, circulating tumor DNA; IHC, immunohistochemistry; MB, megabase; MSI, microsatellite instability; NA, not available; PD-L1, programmed death-ligand 1; PET, positron emission tomography; TMB, tumor mutational burden.

Our index patient is a 64-year-old never-smoker Caucasian woman diagnosed with having a T3 (two separate nodules) N0M0 NSCLC (adenocarcinoma) at 59 years old and underwent a curative right upper lobe (RUL) lobectomy. No molecular profiling was performed at the time owing to early stage disease. She received four cycles of cisplatin/pemetrexed chemotherapy followed by regular surveillance computed tomography (CT) scans. After 4 years, she presented with chest pain. A 3.3 cm left breast mass and confluent left axillary lymphadenopathy were found on a CT scan, and the biopsy result revealed moderately metastatic adenocarcinoma with mucinous features with immunohistochemistry staining consistent with lung primary. Results of complete staging with magnetic resonance imaging of the brain and positron emission tomography scan revealed only the left breast, axillary, and mediastinal lymphadenopathy (Fig. 1*A*). Result of initial limited molecular profiling revealed *ROS1* fluorescence in situ hybridization positivity. The patient was assumed to have developed metastases from the previous RUL adenocarcinoma and was started on entrectinib 600 mg daily. The patient transferred care to our institution, and results of comprehensive tissue DNA and RNA NGS (Caris Life Science, Phoenix, AZ) detected a *CD74-ROS1* fusion variant (C6, R34). A *CHEK2* S5fs∗ mutation was detected in the tumor (allele frequency [AF]: 42%). Plasma genotyping (Foundation Medicine, Boston, MA) revealed *CHEK2* S5fs∗54 (c.14_20delCGGATGT) at an AF of 50.2% consistent with a heterozygous germline mutation (Fig. 1*B*). Family history revealed one cousin with breast adenocarcinoma with the same *CHEK2* mutation and her daughter was found to be a *CHEK2* mutation carrier. Genetic testing was suggested to our patient and her siblings because there is a 50% chance of her siblings being carriers which would increase their children’s chance of cancer.

Because of the atypical presentation of *CD74-ROS1* NSCLC with the left breast and lymphadenopathy without a clear primary tumor, we requested retrospective DNA and RNA NGS of the previously resected RUL sample (Caris Life Science, Phoenix, AZ) which revealed *EGFR* E746 _S752 delinsV (AF: 16%) and no evidence of *CD74-ROS1*. *CHEK2* S5f∗ mutation AF was 81%. Thus, our patient number (#)1 has two separate NSCLC primaries separated by approximately 4 years.

We then further identified a second 70-year-old female Caucasian never-smoker patient with a germline *CHEK2* T367fs∗15 (1100delC) mutation. The patient was diagnosed with having a 1.3 cm left upper lobe well-differentiated NSCLC with *KRAS* G12C when she was 66 years old. She underwent left upper lobe lobectomy and continues having CT surveillance without recurrence. In addition, her *CHEK2* mutation (AF: 69%) was confirmed to be germline by Ambry Genetics (Aliso Viejo, CA) hereditary cancer plasma genotyping assay. Details of both cases are summarized in Figure 1*C*.

**Figure 2:**
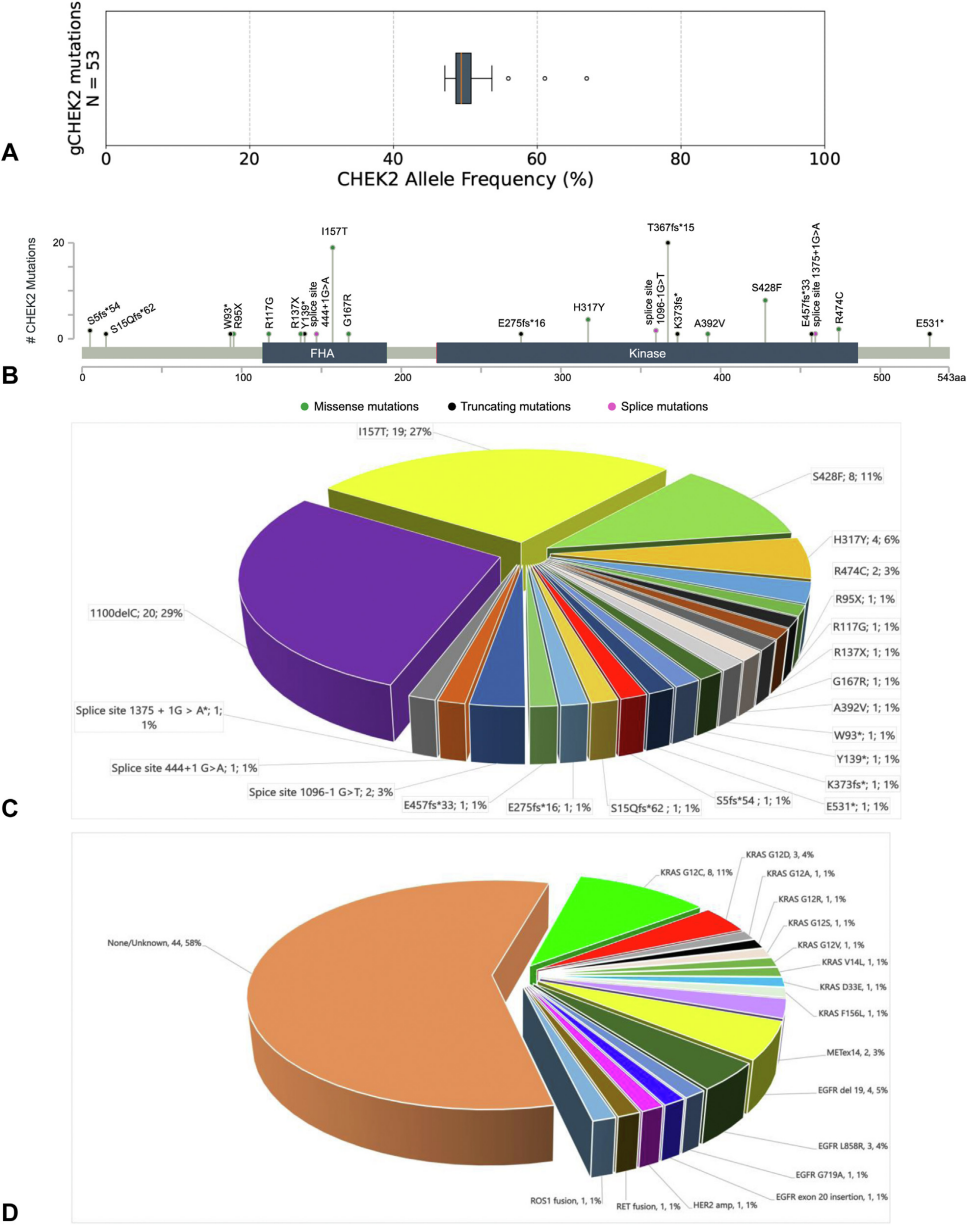
(*A*) The median AF of germline *CHEK2* mutations in the Foundation Medicine genomic database revealing median AF of 49% (IQR: 49%–51%). (*B*) Lollipop diagram of the germline *CHEK2* mutations identified in this study among 70 total NSCLC cases. (*C*) Pie chart of the types and frequency of the germline *CHEK2* mutations in this study among 70 total *gCHEK2m* NSCLC cases. (*D*) Pie chart of the driver alterations identified with germline *CHEK2* mutations in this study among 70 total *gCHEK2m* NSCLC cases. AF, allele frequency; *gCHEK2m*, germline *CHEK2* mutation; IQR, interquartile range.

These two cases prompted us to further investigate the incidence and correlation between *CHEK2* mutations and actionable driver alterations in NSCLC by querying the Foundation Medicine genomic database. Of 6101 patients with unique NSCLC profiled by plasma genotyping, 53 cases (0.87%) of *gCHEK2m* were identified (male-to-female ratio, 49%:51%; median age = 75 y). Ten (excluding our case #1) unique *CHEK2* germline mutations were identified, including most often I157T and T367fs∗15 (each 36% of cases). The median *gCHEK2m* AF was 49% (interquartile range: 49%–51%) (Fig. 2*A*). There was one *CHEK2* mutation (E457fs∗33) predicted to be homozygous (AF = 67%). Among these 53 *gCHEK2m*, 49 occurred in samples with detectable circulating tumor DNA (ctDNA). Actionable driver alterations (*KRAS* G12C [n = 6], *MET*ex14 [n = 2], *EGFR* exon 19 deletion [n = 1], *EGFR* G719A [n = 1], *EGFR* exon 20 insertion [n = 1], *ERBB2* amplification [n = 1], *RET* fusion [n = 1]) co-occurred in the ctDNA in 13 of 49 (26.5%) patients with *gCHEK2m*. In addition, six potentially actionable co-occurring *KRAS* mutations were identified (G12D [n = 3], G12A [n = 1], G12R [n = 1], G12S [n = 1]).

Finally, survey of the literature identified two case reports and one family with homozygous *CHEK2* mutation with multiple cancers, including NSCLC,^3^, ^4^, ^5^ and two germline surveys of *CHEK2* mutations in NSCLC.^6^^,^^7^ Tian et al.^6^ identified seven *gCHEK2m* among 1764 Chinese patients (0.40%) with NSCLC. Liu et al.^7^ identified four among *gCHEK2m* 1026 Chinese patients (0.39%) with NSCLC. Combining the two Chinese survey studies and the Foundation Medicine database, *gCHEK2m* were found in 0.72% (64 of 8891) of NSCLC. Overall, literature survey identified 15 additional NSCLC *gCHEK2m* cases, 11 in the Chinese surveys and four additional reported cases (Table 1). Furthermore, six of the 15 cases had actionable driver alterations (either *EGFR* or *KRAS* G12C mutation) (Table 1). Overall, a total of 70 *gCHEK2m* were identified (53 in the Foundation Medicine data set, 15 in the literature, and two reported herein), representing 21 unique mutations, the most common of which were T367fs∗15 (29%) and I157T (27%) (Fig. 2*B* and *C*). Among the 70 *gCHEK2m*, 54.3% were amino acid substitutions (point mutations), 40.0% were frameshift mutations, and 5.7% were splice site mutations. Of these 70 total cases assessed, a total of 29 (41.4%) potentially actionable driver alterations were identified; *KRAS* G12C mutation (27.6%) was the most common and *KRAS* G12A/C/D/R/S/V mutations constituted an additional 51.7% of driver-positive cases (Fig. 2*D*). We did not consider *KRAS* V14L, D33E, and F156L actionable alterations currently.

**Table 1:** Survey of Literature of Germline *CHEK2* Mutation and NSCLC

#	Case # in the Reference	Sex	Age	NSCLC Histology	*CHEK2* Mutation	Actionable Driver Mutation Identified	Smoking Status	Reference [#]
1		F	62	Adenocarcinoma with solid and cribriform patterns (right lung); papillary adenocarcinoma (left lung)	E275fs∗16	KRAS G12V (right lung), KRAS G12C (left lung)	FS	Carey et al.^3^^,^^a^
2		F	55	AdenoCA (with a simultaneous breast adenocarcinoma)	G167R	None (STK E65D)	NS	Di Federico et al.^4^
3		F	60	Multifoci adenoCA	R474C (homozygous)	EGFR L858R, multifoci (not all tested)	NS	Kukita et al.^5^
4		M	60	Multifoci adenoCA	R474C (homozygous)	Not reported	FS	Kukita et al.^5^
5	Case 2	M	71	SqCC	H317Y	None	NR	Tian et al.^6^
6	Case 16	F	66	AdenoCA	H317Y	None	NR	Tian et al.^6^
7	Case 30	F	66	AdenoCA	H317Y	EGFR S752_I759del, EGFR amplification	NR	Tian et al.^6^
8	Case 59	F	71	AdenoCA	H317Y	EGFR L858R	NR	Tian et al.^6^
9	Case 34	F	55	AdenoCA	Y139∗	EGFR E746_A750del	NR	Tian et al.^6^
10	Case 47	F	75	AdenoCA	CHEK2 1375+1G>A∗	None	NR	Tian et al.^6^
11	Case 66	F	68	AdenoCA	CHEK2 S15Qfs∗62	EGFR L858R, EGFR amplification	NR	Tian et al.^6^
12	Case 7	M	54	AdenoCA	R95X	NR	NS	Liu et al.^7^
13	Case 8	M	75	Large cell	R137X	NR	FS	Liu et al.^7^
14	Case 9	F	66	AdenoCA	K373Fs	NR	NS	Liu et al.^7^
15	Case 30	F	77	AdenoCA	IVS1096-1G>C	NR	NS	Liu et al.^7^

#, number; adenoCA, adenocarcinoma; ctDNA, circulating tumor DNA; F, female; FS, former smoker; g*CHEK2*, germline *CHEK2*; M, male; NR, not reported; NS, not significant; SqCC, squamous cell carcinoma.

a This case was not included in the 53 cases of Foundation Medicine database owing to low level of ctDNA that did not pass internal quality control metrics of Foundation Medicine to call *gCHEK2* mutation. The *gCHEK2* mutation in Carey et al.^3^ was confirmed by Ambry Genetics.

## Discussion

We believe that this is the most comprehensive study of *gCHEK2m* in NSCLC to date. Combined survey of plasma samples from a commercial genomic database and survey of the literature revealed that *gCHEK2m* are rare (<1%) in NSCLC. Furthermore, the germline alterations occurred throughout the entire *CHEK2* gene and consisted of amino acid changes, frameshift mutations, and splice site mutations. Importantly, more than 40% NSCLC with *gCHEK2m* harbored actionable drivers with proven therapeutics treatments that could potentially extend to 51% if considering other *KRAS* G12 isotype mutations.

The *CHEK2* mutation 1100delC and *CHEK2* I157T mutations associated with hereditary breast cancer are the two most common *CHEK2* mutations identified in NSCLC.^1^^,^^8^ *CHEK2* S5Lfs∗54 from our patient #1 is extremely rare, limited information has been published in the literature listed by the National Library of Medicine, and it is only reported four times in the National Library of Medicine database by commercial sequencing companies,^9^ including in a survey of hereditary genetic mutations potentially predisposing to pancreatic adenocarcinoma.^10^ This frameshift mutation resulted in a deletion of seven bases with a frameshift starting at the fifth amino acid and resulted in a stop codon downstream by 54 bases (S5fs∗54), and it is expected to result in loss of function by premature protein truncation or nonsense-mediated mRNA decay and is considered pathogenic.

The initial NSCLC of our patient #1 was found to harbor *EGFR* exon 19 deletion mutation in retrospect. At the time of diagnosis, the standard of care was four cycles of adjuvant chemotherapy. The recently published ADAURA trial indicated that 3 years of adjuvant osimertinib significantly improved 2-year disease-free survival rate to a similar extent with or without adjuvant cisplatin-based chemotherapy.^11^ Thus, our patient #1, if diagnosed in 2021, could potentially have avoided adjuvant cisplatin-based chemotherapy.^12^ We wonder if the cisplatin-based chemotherapy may have promoted the genesis of the second *ROS1+* NSCLC given that cisplatin induces DNA strand breaks and the *gCHEK2m* reduces the corrective response to DNA breaks.

Plasma genotyping will identify *gCHEK2m* at AF ~50% as shown in Figure 2*A* while tumor genotpying the AF of *gCHEK2m* could vary widely though a high AF should alert clinicians to the potential presence of *gCHEK2m*. One of the limitations of this study is that not many patients with NSCLC undergo plasma NGS genomic profiling, not all plasma NGS genomic profiling assays include *CHEK2*, and not all NGS assays sequence the whole *CHEK2* gene. Thus, the frequency of *gCHEK2m* among NSCLC may be underestimated in the literature. Given the predominance of *EGFR* mutations among Asians, a comprehensive NGS may not be routinely performed. Indeed, no *KRAS* mutations were reported among Chinese patients with NSCLC with *gCHEK2m.* Furthermore, actionable driver alterations could only be detected in patients who are tumor ctDNA “shedders,” which is often a surrogate of tumor burden. For example, in our patient case #1, the *ROS1* fusion was not detected in the plasma as she was already receiving effective treatment at the time of plasma genotyping.

In summary, *gCHEK2m* are rare in NSCLC but they span the entire *CHEK2* gene with 21 unique mutations identified comprising point mutations, frameshift mutations, and splice site mutations. At least 40% of these NSCLC with *gCHEK2m* harbor diverse actionable driver alterations. Whether there should be increased surveillance for lung cancer in *gCHEK2* carriers remained to be determined given that NSCLC has rarely been reported in *gCHEK2* carriers. Furthermore, treatment with inhibitor of PARP in combination with targeting the underlying actionable driver mutation in these rare *gCHEK2m+* NSCLC warrants future investigations.

## CRediT Authorship Contribution Statement

**Shannon S. Zhang:** Conceptualization, Data curation, Formal analysis, Investigation, Methodology, Resources, Software, Validation, Writing—original draft, Writing—review and editing.

**Jessica K. Lee:** Investigation, Data curation, Formal analysis, Methodology, Resources, Software, Validation, Visualization, Writing—original draft, Writing—review and editing.

**Hanna Tukachinsky:** Investigation, Data curation, Formal analysis, Methodology, Resources, Software, Validation, Writing—original draft, Writing—review and editing.

**Alexa B. Schrock:** Investigation, Data curation, Formal analysis, Methodology, Resources, Software, Validation, Writing—original draft, Writing—review and editing.

**Misako Nagasaka:** Investigation, Data curation, Formal analysis, Writing—original draft, Writing—review and editing.

**Sai-Hong Ignatius Ou:** Conceptualization, Data curation, Formal analysis, Investigation, Methodology, Project administration, Resources, Supervision, Validation, Visualization, Writing—original draft, Writing—review and editing.
